# Duration of the flaxseed diet promotes deposition of n-3 fatty acids in the meat and skin of Peking ducks

**DOI:** 10.29219/fnr.v63.3590

**Published:** 2019-12-05

**Authors:** Muhammad Suhaib Shahid, Yuqin Wu, Zhibin Xiao, Tausif Raza, Xiaoyu Dong, Jianmin Yuan

**Affiliations:** State Key Laboratory of Animal Nutrition, College of Animal Science and Technology, China Agricultural University, Beijing, China

**Keywords:** flaxseed, LPIN-1, FADS2, DHA, meat, duck

## Abstract

**Background:**

Polyunsaturated fatty acids (PUFA), particularly n-3, have beneficial effects on human health, and for this reason foodstuffs with increased content of n-3 PUFA are now very common and widely available.

**Design:**

This study was conducted to investigate the effect of the duration of a flaxseed diet on Peking duck’s growth performance, antioxidant status, gene expression, and fatty acid profile of the meat. A total of 792 12-day-old white Peking ducks were divided into four groups. In the control group, animals were provided with a basal diet. In the three experimental groups, animals were fed a 10% flax seed diet with vitamin E at 13, 23, and 33 days of age for 30, 20, and 10 days, respectively.

**Results:**

The growth performance of the ducks decreased with flaxseed diet’s duration. Both body weight and body weight gain decreased linearly while Feed conversion ratios (FCR) increased in the group of ducks fed flaxseed compared to control ducks. Serum triglycerides (TG), very low density lipoprotein (VLDL), low density lipoprotein cholesterol (LDL-C), and aspartate aminotransferase (AST) linearly decreased while high density lipoprotein cholesterol (HDL-C) and lipopolysaccharide (LPS) levels increased by feeding flaxseed up to 30 days. The expression of lipin-1 gene (LPIN-1) and fatty acid desaturase 2 (FADS2) linearly increased in ducks fed flaxseed for 30 days. Linolenic acid (n-3) and its long-chain metabolites like eicosatetraenoic acid (ETA), eicosapentaenoic acid (EPA), docosahexaenoic acid (DHA), and total n-3 fatty acids (FA) linearly increased while the ratio of n-6 to n-3 was reduced with increased duration of flaxseed supplementation.

**Conclusion:**

Overall, we found that increasing the duration of flaxseed diet with vitamin E for more than 10 days had a mild adverse effect on duck’s growth performance but enrichedits meat with long-chain PUFA and decreased the n-6 to n-3 ratio, providing quality meat for health-conscious consumers. A period of 20 days is good for producing n-3 enriched Peking duck meat and skin.

## Popular scientific summary

- Flaxseed was fed to ducks for 0, 10, 20, and 30 days.
- Flaxseed decreased growth performance and abdominal fat.
- A 30-d feeding improved meat quality and increased expression of Lipin-1 and FADS2 genes.
- Flaxseed fed over a period of 20 days enriched Peking duck’s meat and skin with linolenic acid, DHA, and EPA, making it suitable for health conscious consumers.

In humans, high ratios of n-6/n-3 polyunsaturated fatty acids (PUFA) increase the risk of obesity and cardiovascular diseases (1). Because n-3 PUFA has beneficial effects on cardiovascular and neurological health, inflammation and immunity (2), it is possible to improve human nutrition by supplying C18:3 n-3 enriched foods (3). Chickens cannot have the capacity to synthesize n-3 PUFA, so these must be delivered in their feed.

Most lipids are ingested in the form of TG, but must be broken down into FA, and transported by fatty acid-binding proteins (FABP) or fatty acid transport protein (FATP) across the plasma membrane in the intestine (4, 5). In the liver, FA were metabolized by various genes like Lipin 1 (LPIN-1) and WD and tetra-Trico peptide repeats 1 (WDTC1) (6), n-3 FA were converted to DHA and EPA by FADS2 (7), and β-oxidized by the PPAR-α transcriptional factor in peroxisome and mitochondria (8). Previous studies reported that dietary n-3 increased the expression of FADS2 and LPIN1 genes in broilers (6, 9, 10).

Lipoprotein lipase (LPL) takes part in the clearance of chylomicrons arising from dietary fat preventing it to enter the tissue and inhibiting TG accumulation (11). LPS is the part of the cell wall of Gram-negative bacteria that enter the circulation from the intestinal lumen in response to a high-fat diet (12).LPS decreased the LPL activity, thus interrupting the TG removal from circulation (13).

Flaxseed (*Linum usitatissimum*), is popular for its high content of n-3 and widely used as a source of n-3 (14). Recently, flaxseeds were also incorporated into food with other nutraceuticals to improve the nutritional quality of food (15). Studies on human and animals prove that consumption of flaxseed decreased the progression of atherosclerosis, decreased blood pressure, improved brain function, and provided protection against cancer (16). In poultry, it enhances the n-3 content in eggs and meat, providing meat enriched in n-3 PUFA for which many positive health effects have been described (17). However, the presence of anti-nutritional factors like mucilage, linatinedi-peptide (a vitamin B6 antagonistic), cyanogenic glycosides, trypsin inhibitor, and phytic acid can limit its use in poultry feed (18). These anti-nutritional factors in flaxseed decreased body weight and increased the feed conversion ratio (FCR) in different species of birds (19, 20). In addition, feed enriched with PUFA can be prone to oxidation, causing oxidative stress in animals (21). A study showed that increasing the content of flaxseed up to 15% decreased the acceptability of broiler meat nuggets due to the lipid peroxidation of PUFAs (22). Previous studies also reported that flaxseed can enhance activities of ALT and AST (23). Therefore, antioxidants are added to diets of chickens to prevent oxidative damage (24). Combining n-3 FA with vitamin E could lower lipid oxidation activity and increase antioxidant status in plasma of broilers (25), without a negative impact on the physicochemical and sensory attributes of meat (19).

Roast Peking duck is a famous dish in the world. The skin comprises most of the roast Peking duck; however, it is normally enriched with n-6 FAs, which is not beneficial for people’s health (26). Although many researchers studied the effect of duration of feeding flaxseed in broilers and pigs (27, 28), however, little attention was focused on the effect of the duration of flaxseed feed on Peking ducks. Therefore, the present study was conducted to investigate the effect of feeding period for flaxseed on performance, antioxidant status, n-3 content, and expression of genes related to fatty acid transport and metabolism of the Peking duck.

## Materials and methods

All animal-related work was approved by the China Agricultural University Animal Care and Use Committee. All protocols and procedures were performed according to the Chinese Regulations for Laboratory Animals.

### Bird husbandry

A total of 792 12-day-old white Peking ducks were divided into four groups. Every treatment group had six replicates with 33 ducks each. The control group (D) was given a basal diet from the 13th to the 42nd day. Groups on a flaxseed diet were provided a 10% flaxseed diet starting from the 13th, 23rd, and 33rd day of age for 30, 20, and 10 days, respectively. The composition of diets and nutrient analysis of diets and flaxseed are shown in Tables 1 and 2. Feed and water supply to the ducks were given *ad-libitum*.

**Table 1:** Ingredients composition of ducks diets

Ingredient basis %	Control diet	Flaxseed diet
Corn	65.50	–
Wheat 13%	–	55.74
47% Soybean meal	29.00	15.50
Soy oil	1.8	–
Flaxseed	–	10.00
Barley	–	15.00
Calcium carbonate	1.58	1.40
Monocalcium phosphate 21%	1.30	1.30
Salt	0.36	0.36
Methionine	0.11	0.11
Choline chloride, 60%	0.10	0.10
Lysine HCl	0.03	0.25
Vitamin premix^1^	0.02	0.02
Vitamin E (50%)		0.02
Mineral premix^2^	0.20	0.20
Total	100.00	100.00

1 The vitamin premix provided the following per kilogram of diet: vitamin A, 10,000 IU; vitamin D_3_, 2,400 IU; vitamin E, 20 IU; vitamin K_3_, 2.00 mg; thiamin, 2.00 mg; riboflavin, 6.40 mg; pyridoxine, 3.00 mg; VB_12_, 0.02 mg; folic acid, 1.00 mg; pantothenic acid, 10.00 mg; nicotinic acid, 30.00 mg; biotin, 0.10 mg.

2 The mineral premix provided following per kg of diet: Cu, 8 mg; Fe, 80 mg; Zn, 60 mg; Mn,100 mg; I, 0.35.

**Table 2:** Nutrient analysis of duck diets

Chemical composition	Control diet	Flaxseed diet	Flaxseed
MEn Poultry (Kcal/kg)	2950.0	2950.0	
Protein %	19.0	19.0	
Lysine %	1.01	1.01	
Methionine %	0.40	0.40	
M+C	0.69	0.72	
Threonine %	0.73	0.64	
Ca%	0.90	0.90	
Available phosphorus%	0.39	0.39	
Fatty acids composition %	Control diet	Flaxseed diet	
Myristic acid	0.4	0.2	0.1
Palmitic acid C16:0	9.8	8.4	6.7
Margaric acid C17:0	0.3	0.2	0.1
Palmitoleic acid C16:1	0.12	0.09	0.08
Oleic acid C18:1n9c	29.02	16.08	20.5
Arachidic acid C20:0	0.14	0.08	0.09
Linoleic acid C18:2n6	35.66	24.21	14.2
Eicosadienoic acid	N.D	N.D	N.D
Dihomo-γ-linolenic acid	N.D	N.D	N.D
Alpha Linolenic acid n3	3.22	33.42	54.00
ETA C20:3 n3	N.D	N.D	N.D
EPA C20:5 n3	N.D	N.D	N.D
DHA C22:6 n3	N.D	N.D	N.D

*Note*: ETA = Eicosatetraenoic acid, EPA = Eicosapentaenoic Acid, DHA = docosahexaenoic acid, N.D = not detected.

### Measurements

#### Performance parameters

Body weight (BW) and feed intake (FI) were recorded at the end of the experiment. Feed conversion ratios, survival rate, and European index were calculated.

#### Sampling and preparation

At the end of the experiment (day 30, when ducks’ age was 42 days), one bird with a BW similar to the mean BW of the replicates in its group was selected for each treatment. A blood sample was collected from the wing’s vein in a non-heparinized tube and centrifuged at 4,000 *g* for 15 min. Serum was separated and stored at −20°C for further analysis. After bleeding, birds were killed by stunning. Jejunum and liver were removed manually. A 0.5-cm long section from the middle segment of the jejunum was collected from six birds per pen, flushed with cold PBS, and then frozen using liquid nitrogen and stored at –80°C for further analysis.

#### Carcass traits

Carcass traits were measured according to Chinese performance terms and measurement method for poultry (29). Abdominal fat, subcutaneous fat, skin, and breast meat were removed manually from the carcass and weighted. Carcass traits such as skin, abdominal fat, and breast muscle were weighed. Their weights are expressed as relative weights (part weight/live weight)×100.

#### Meat quality

pH was measured 24 h post-mortem using a portable pH/°C measuring instrument, Testo 206-pH2 and pH2 piercing probe head for semi-solid substances (Testo GmbH & Co., Lenzkirch, Germany). Drip loss from the breast meat was determined as described earlier (30).

#### Serum biochemical and antioxidant indices

The serum indices, namely TG, VLDL, LDL-C, HDL-C, alkaline phosphatase (ALP), AST, alanineamino transferase (ALT), LPL and LPS and were measured using commercially available kits (Nanjing Jiancheng Bioengineering Institute, China) following the manufacturer’s instructions.

#### Determination of oxidative parameters in breast muscle, liver, and jejunal mucosa

The frozen breast muscle, jejunal mucosa, and liver pieces were homogenized in 0.86% (w/v) sodium chloride solution (0.9 mL added per gram of tissue) at 4°C using an Ultra-Turrax T8 homogenizer (IKA Labortechnik, Staufen, Germany) for 1–2 min at 3,000–5,000 r/min. The homogenates were centrifuged (4,000 *g* for 5 min at 4°C) and the supernatants were used to determine the indices of oxidative stress. The oxidative indicators, SOD and MDA, in the breast muscle, liver, and jejunal mucosa were quantified using assay kits (Nanjing Jiancheng Bioengineering Institute, China) following the manufacturer’s instructions (31).

#### RNA extraction and reverse transcription

Total RNA of liver samples was extracted using Trizol Reagent (Invitrogen Biotechnology Inc., Carlsbad, CA) according to the manufacturer’s protocol. Sequences encoding the genes for duck Δ6-desaturase or FADS2, lipin1 gene (LPIN1), lipin 2 gene (LPIN2), L-FABP, peroxisome proliferator activated receptors alpha (PPAR-α), WD and tetra-Trico peptide repeats 1(WDTC1), and FATP are shown in Table 3. Real-time PCR to measure the expression of lipid metabolism-related genes in the liver was carried out using SYBR Premix Ex Taq (TliRNaseH Plus) (Takara Biotechnology Inc., Osaka, Japan) on an ABI 7,500 real-time PCR System (Applied Biosystems, Foster City, CA). A reaction volume of 20 μL of mixture contained 10 μL SYBR Premix Ex Taq (TliRNaseH Plus) (2×), 0.4 μL ROX reference dye-II (50×), 0.4 μL each of forward and reverse primer (Table 2), 6.8 μL of easy dilution, and 2 μL of cDNA template. The optimized protocol for all the genes was 95°C for 30 s followed by 40 cycles of 95°C for 5 s and 60°C for 34 s. All measurements were carried out in triplicate and the average values were obtained. Real-time PCR efficiency for each gene was calculated based on the slope of the cDNA relative standard curve that was formulated using a pooled sample. The specificity of the PCR products was evaluated by the analysis of the melting curve. Results of relative mRNA expression genes were calculated using the 2^−ΔΔCt^ method (32).

**Table 3:** Sequences of primer pairs of mRNA

mRNA	FORWARD	REVERSE
*LPIN 1*	TCATCCAGAGTGCTCCTGTG	TCTGGCTTATTTGCCTCAGC
*LPIN 2*	CAGGATGGCACATACCAGTG	TCCATTGTCACCCAGTTTCA
*FADS2*	TCTCCTCCTTGCATTCATCC	ACTTGTGGACGAGGTGGTTC
*FATP*	TCGCAGTGTATGGAGTGGAA	GGAGGCAGCACCTTCTGTAG
*L-FABP*	CACTGCCCCCACACTGCGTT	CGTCACCACAAAGTCGTCTCCT
*PPAR-α*	CAGAGTCATCCTTGCAGG	GTCAAGATTGGAGAAGCC
*WDTC1*	TATCAACGCAGCCCTCTTCT	CCTCAGCACCATCTCATCCT
β-actin	AAATCAAGATCATTGCCCCACCT	AGGGGTGTGGGTGTTGGTAA

*Note*: LPIN 1: lipin 1 gene encoding lipin 1 protein; LPIN 2: lipin 2 gene encoding lipin 2 protein; FADS2:fatty acid desaturase 2 encoding Δ-6 desaturase; FATP: fatty acid transport protein; FABP: fatty acidbinding protein; PPAR-α: peroxisome proliferatoractivated receptors alpha;WDTC1: WD and tetratricopeptide repeats 1.

#### Fatty acid analysis

For fatty acids (FA) analysis, 100 mg of feed, minced meat, and skin were weighed and then extracted, saponified, and methylated according to a previously reported procedure of (33). The FA methyl esters were subjected to gas chromatography mass spectrometry (SCION-456, China) for fatty acid separation. The column measurements were (30 m × 0.32 mm × 0.25 m). The carrier gas used was Helium at a flow rate of 1.5 cm^3^/min. The column, detector and injector temperatures were set at 195, 250, and 225°C, respectively. FAs were identified comparing their retention times with authentic standards.

### Statistical analysis

Data were analyzed as a completely randomized design using SPSS 20.0 (34). Linear and quadratic relations were used to check the significance. Post hoc significance was set at *P* < 0.05.

## Results

### Performance

Feeding period of flaxseed linearly decreased body weight, FCR, European index, and body weight gain (*P* < 0.05) (Table 4). FI and survival rate were not significantly affected. Body weight decreased by 1.99, 5.91, and 6.16% for ducks fed flaxseed for 10, 20, and 30 days (*P* < 0.05), respectively and body weight gain decreased by 2.61, 9.24, and 9.04% for ducks fed flaxseed for 10, 20, and 30 days as compared to control ducks (*P* < 0.05). Feed conversion rate increased by 6.64, 15.04, and 18.14% in ducks fed flaxseed for 10, 20, and 30 days as compared to control ducks (*P* < 0.05).

**Table 4:** Effect of supplemental different days of flaxseed on performances of Peking ducks

Supplemental Days	BW (kg) mean ± SE	FI (kg)	FCR	SR (%)	EI	BWG (kg)
30	3.38 ± 0.01^b^	6.00 ± 0.10	2.67 ± 0.02^a^	97.48 ± 0.93	413.20 ± 3.13^b^	2.33 ± 0.01^c^
20	3.39 ± 0.04^b^	6.00 ± 0.13	2.60 ± 0.01^a^	100.00 ± 0.00	427.00 ± 7.32	2.33 ± 0.04^c^
10	3.54 ± 0.03^a^	5.89 ± 0.14	2.41 ± 0.03^b^	97.48 ± 0.93	482.11 ± 9.76^a^	2.50 ± 0.03^b^
0	3.61 ± 0.02^a^	5.72 ± 0.05	2.26 ± 0.02^c^	97.98 ± 0.64	530.50 ± 6.78^a^	2.57 ± 0.12^a^
*P* value						
ANOVA	<0.001	0.215	<0.001	0.073	<0.001	<0.001
LINEAR	<0.001	0.061	<0.001	0.761	<0.001	<0.001
QUADRATIC	0.305	0.368	0.003	0.183	0.026	0.237

*Note*: A = 30 days, B = 20 days, C = 10 days, D = 0 days; data presented are mean; *n* = 6; means in the same column are significantly different at *α* = 0.05; BW = body weight, FI = feed intake, FCR = feed conversion ratio, SR = survival rate, EI = European Index, BWG = body weight gain.

### Carcass characteristics

The relative weights of the skin, breast muscle, and abdominal fat were significantly affected by the duration of the flaxseed diet (*P* < 0.05) (Table 5). There were linear correlations between the duration of the flaxseed diet and the relative weight of skin and abdominal fat. Breast muscle’s relative weight was quadratically affected by flaxseed duration (*P* < 0.05). Abdominal fat decreased linearly by 10.4, 25.6, and 34.4% (*P* < 0.05) in ducks fed flaxseed for 10, 20, and 30 days, respectively, as compared to control ducks.

**Table 5:** Effect of supplemental different days of flaxseed on carcass traits and meat quality of Peking ducks

Supplemental days	Relative weight (part weight/live weight × 100)			Meat quality	
	Skin	Breast meat	A.Fat	pH	Drip loss (%)
30	22.28 ± 0.51^c^	8.37 ± 0.35^c^	0.82 ± 0.04^b^	5.61 ± 0.02	1.67 ± 0.21^b^
20	23.55 ± 0.63^b^	10.70 ± 0.35^a^	0.93 ± 0.04^b^	5.63 ± 0.04	2.38 ± 0.20^a^
10	25.60 ± 0.64^b^	9.61 ± 0.10^b^	1.12 ± 0.09^a^	5.61 ± 0.01	2.40 ± 0.30^a^
0	26.23 ± 0.33^a^	7.97 ± 0.60^c^	1.25 ± 0.05^a^	5.64 ± 0.02	2.48 ± 0.12^a^
*P* value					
ANOVA	0.016	0.014	0.001	0.840	0.045
LINEAR	0.002	0.388	0.001	0.499	0.017
QUADRATIC	0.760	0.003	0.938	0.945	0.150

*Note*: A = 30 days, B = 20 days, C = 10 days, D = 0 days; A.Fat = abdominal fat; data presented as mean; *n* = 6;means in the same column are significantly different at *α* = 0.05.

### Quality of breast muscle

Drip loss was significantly affected by flaxseed duration (*P* < 0.05) (Table 5). Drip loss of breast meat decreased linearly by 3.22, 4.03, and 32.66% for ducks fed flaxseed for 10, 20, and 30 days compared to the control (*P* < 0.05). The pH of breast meat was not significantly affected by duration of the flaxseed diet (*P* < 0.05).

### Anti-oxidation character

Breast muscle, liver, and jejunal mucosa SOD activity were not significantly affected by duration of the flaxseed diet (*P* < 0.05) (Table 6). However, MDA decreased linearly with duration of the flaxseed diet in the breast muscle, liver, and jejunal mucosa (*P* < 0.05).

**Table 6:** Effect of supplemental different days of flaxseed on fatty acid metabolism, endocrine hormones and oxidative indices of Peking ducks

	Supplemental days	30	20	10	0	*P*	Linear	Quadratic
Serum	TG (mmol/L)	0.69 ± 0.06^b^	0.76 ± 0.07^b^	1.23 ± 0.19^a^	1.44 ± 0.21^a^	0.005	0.001	0.653
	LDL-C (mmol/L)	1.92 ± 0.05^c^	2.23 ± 0.03^b^	2.47 ± 0.05^a^	2.50 ± 0.03^a^	<0.001	<0.001	0.003
	VLDL-C (mmol/L)	1.03 ± 0.01^d^	1.17 ± 0.03^c^	1.31 ± 0.03^b^	1.59 ± 0.04^a^	<0.001	<0.001	0.038
	HDL-C (mmol/L)	1.48 ± 0.03^a^	1.36 ± 0.02^b^	1.24 ± 0.03^c^	1.06 ± 0.06^d^	<0.001	<0.001	0.448
	LPS (U/L)	60.16 ± 4.01^a^	50.53 ± 3.27^b^	53.25 ± 1.30^ab^	37.56 ± 1.60^c^	<0.001	<0.001	0.289
	LPL (U/mL)	3.07 ± 0.05^a^	2.77 ± 0.19^a^	2.47 ± 0.28^a^	1.49 ± 0.26^b^	<0.001	<0.001	0.127
	ALP (U/L)	1112.60 ± 62.24	1055.68 ± 96.14	1130.75 ± 1.16	1195.24 ± 84.95	0.761	0.442	0.517
	ALT (U/L)	19.48 ± 1.67	18.38 ± 1.48	18.65 ± 0.97	19.58 ± 1.55	0.914	0.931	0.489
	AST (U/L)	52.46 ± 1.48^b^	69.33 ± 18.03^ab^	38.49 ± 4.18^b^	105.25 ± 17.50^a^	0.009	0.037	0.065
Meat	MDA (nmol/mgprot)	3.71 ± 0.01^c^	3.72 ± 0.01^c^	3.87 ± 0.03^b^	4.35 ± 0.03^a^	<0.001	<0.001	<0.001
	SOD (nmol/mgprot)	210.93 ± 0.38	211.78 ± 0.79	210.33 ± 1.19	209.93 ± 0.42	0.369	0.209	0.424
Liver	MDA (nmol/mgprot)	3.30 ± 0.20^b^	3.53 ± 0.25^b^	3.61 ± 0.60^b^	5.28 ± 0.28^a^	0.004	0.002	0.067
	SOD (nmol/mgprot)	209.98 ± 19.25	216.69 ± 12.15	198.66 ± 7.45	181.01 ± 4.55	0.212	0.069	0.330
Jejunum mucosa	MDA (nmol/mgprot)	3.46 ± 0.17^b^	3.67 ± 0.18^b^	3.95 ± 0.20^b^	4.68 ± 0.13^a^	<0.001	<0.001	0.141
	SOD (nmol/mgprot)	211.67 ± 7.02	221.11 ± 9.90	198.25 ± 11.67	199.44 ± 6.76	0.264	0.158	0.654

*Note*: A = 30 days, B = 20 days, C = 10 days, D = 0 days. TG = triglycerides, LDL-C = low density lipoprotein cholesterol, VLDL-C = very low density lipoprotein cholesterol, HDL-C = high density lipoprotein cholesterol, LPS = lipopolysaccharide, LPL = lipoprotein lipase, ALP = alkaline phosphatase, ALT = alanineamino transferase, AST = aspartate aminotransferase, MDA = malondialdehyde, SOD = superoxide dismutase. Data presented as mean; *n* = 6. Means in the same column are significantly different at *α* = 0.05.

### Fatty acids metabolic and endocrine hormone

Serum contents of TG, VLDL, LDL-C, HDL-C, and activity of LPL, AST, LPS were significantly affected by duration of the flaxseed diet, while the activity of ALT and ALP were not significantly affected (*P* < 0.05) (Table 6). Serum HDL-C, LPL, and LPS increased linearly from 0 to 30 days of feeding flaxseed while TG, VLDL, LDL-C, and AST decreased linearly with increased duration of the flaxseed diet (*P* < 0.05).

### Gene expression in liver

The expressions of hepatic genes like WDTC1, PPAR-α, FATP, L-FABP, and LPIN2 were not significantly affected by duration of the flaxseed diet (*P* < 0.05). Only the expressions of LPIN1 and FADS2 were found to linearly increase in ducks depending on the flaxseed duration (*P* < 0.05) (Table 7).

**Table 7:** Effect of supplemental different days of flaxseed on relative gene expression in liver of Peking ducks

Supplemental days	LPIN 1	LPIN 2	FADS2	FATP	L-FABP	PPAR-α	WDTC1
30	6.94 ± 1.03^a^	1.08 ± 0.18	1.15 ± 0.14^a^	0.97 ± 0.13	0.99 ± 0.22	1.03 ± 0.10	1.02 ± 0.20
20	3.41 ± 0.88^b^	1.04 ± 0.41	0.86 ± 0.54^ab^	0.77 ± 0.20	0.50 ± 0.10	0.83 ± 0.08	1.09 ± 0.18
10	2.78 ± 0.89^b^	1.53 ± 0.32	1.02 ± 0.11^b^	0.95 ± 0.12	0.71 ± 0.12	0.90 ± 0.06	1.26 ± 0.16
0	1.23 ± 0.39^b^	2.30 ± 0.43	0.56 ± 0.12^c^	0.99 ± 0.20	0.91 ± 0.16	0.77 ± 0.07	1.19 ± 0.10
*P* value							
ANOVA	<0.001	0.063	0.006	0.785	0.142	0.162	0.762
Linear	<0.001	0.041	0.003	0.769	0.972	0.061	0.374
Quadratic	0.250	0.260	0.444	0.478	0.036	0.666	0.696

*Note*: A = 30 days, B = 20 days, C = 10 days, D = 0 days. LPIN 1: lipin 1 gene encoding lipin 1 protein; LPIN 2: lipin 2 gene encoding lipin 2 protein; FADS2 = fatty acid desaturase 2; FATP = fatty acid transport protein; FABP = fatty acid binding protein; PPAR-α = proxisome proliferation activated factor-α; WDTC1: WD and tetratricopeptide repeats 1. Data are presented as mean; *n* = 6. Means in the same column are significantly different at *α* = 0.05.

### Fatty acid profile of breast muscle and skin

Both in the breast muscle (Table 8) and skin (Table 9), my ristic acid (C14:0), palmitic acid (C16:0), margaric acid (C17:0), total saturated fatty acid (SFA), palmitoleic acid (C16:1), oleic acid (C18:1n9), arachidic acid (C20:0), total MUFA, linoleic acid (C18:2n6), eicosadienoic acid (C20:2 only for skin), dihomo-γ-linolenic acid (C20:3n6), and total n-6 decreased linearly in the 10, 20, and 30 days flaxseed diet compared to the control base diet (*P* < 0.05). Linolenic acid n-3, ETA (C20:4 n3), EPA (C20:5 n3), DHA (C22:6 n3), and total n-3 increased linearly in breast muscle and skin (*P* < 0.05). In breast muscle, DHA increased by 337.58, 773.05, and 1033.33% and total n-3 increased by 377.13, 689.75, and 868.04% for ducks fed flaxseed for 10, 20, and 30 days, respectively, compared to the control (*P* < 0.05). In the skin, DHA increased by 658.97, 1407.69, and 2323.08%, while total n-3 increased by 341.74, 656.86, and 852.08% for ducks fed flaxseed for 10, 20, and 30 days respectively, as compared to the control (*P* < 0.05). The ratio of n-3 to n-6 (n-6:n-3) decreased linearly in breast muscle and skin compared to control ducks (*P* < 0.05).

**Table 8:** Effect of supplemental different days of flaxseed on fatty acid profile of breast muscle of Peking ducks

Supplemental days	30	20	10	0	*P*	Linear	Quadratic
Fatty acids (mg/100g)							
Myristic acid C14:0	35.74 ± 0.39^d^	45.09 ± 0.46^c^	54.30 ± 1.07^b^	85.06 ± 0.48^a^	<0.001	<0.001	<0.001
palmitic acid C16:0	282.39 ± 0.66^d^	295.70 ± 0.94^c^	305.52 ± 0.82^b^	366.35 ± 2.61^a^	<0.001	<0.001	<0.001
Margaric acid C17:0	49.33 ± 0.32^d^	52.65 ± 0.44^c^	62.15 ± 1.82^b^	86.63 ± 0.70^a^	<0.001	<0.001	<0.001
Total SFA	367.47 ± 1.03^d^	393.45 ± 0.93^c^	421.97 ± 3.18^b^	538.05 ± 2.54^a^	<0.001	<0.001	<0.001
Palmitoleic acid C16:1	389.10 ± 2.10^d^	399.30 ± 1.20^c^	425.39 ± 1.95^b^	434.18 ± 0.89^a^	<0.001	<0.001	0.667
Oleic acid C18:1n9c	375.38 ± 3.01^d^	413.63 ± 1.32^c^	451.15 ± 0.82^b^	487.03 ± 0.83^a^	0.007	0.001	0.503
Arachidic acid C20:0	25.39 ± 1.07^d^	42.79 ± 0.61^c^	50.82 ± 0.41^b^	60.76 ± 1.18^a^	<0.001	<0.001	<0.001
Total MUFA	789.87 ± 3.02^d^	855.73 ± 2.42^c^	927.36 ± 2.25^b^	981.96 ± 1.19^a^	<0.001	<0.001	0.025
Linoleic acid C18:2n6	426.64 ± 1.39^c^	465.41 ± 2.92^b^	487.88 ± 1.24^a^	489.36 ± 0.96^a^	<0.001	<0.001	<0.001
Ecosadienioc acid C20:2n6	15.76 ± 0.49	16.02 ± 0.46	15.32 ± 0.47	15.78 ± 0.46	0.767	0.763	0.844
Dihomo-γ-linolenic acid C20:3n6	69.47 ± 0.51^b^	72.76 ± 0.33^a^	74.43 ± 1.19^a^	74.84 ± 0.53^a^	<0.001	<0.001	0.059
Arachidonic acid 20:4n-6	10.19 ± 0.12^c^	11.64 ± 0.52^b^	12.50 ± 0.51^ab^	12.96 ± 0.21^a^	<0.001	<0.001	0.206
Total n6	522.05 ± 1.51^c^	565.85 ± 3.04^b^	590.14 ± 1.89^a^	592.94 ± 0.70^a^	<0.001	<0.001	<0.001
Linolenic acid n3	339.76 ± 0.87^a^	278.53 ± 2.49^b^	167.40 ± 0.33^c^	35.61 ± 0.42^d^	<0.001	<0.001	<0.001
ETA C20:4 n3	12.44 ± 0.66^a^	8.56 ± 0.33^b^	5.37 ± 0.25^c^	0.40 ± 0.03^d^	<0.001	<0.001	0.180
EPA C20:5 n3	18.35 ± 0.37^a^	15.93 ± 0.23^b^	11.59 ± 0.25^c^	2.52 ± 0.10^d^	<0.001	<0.001	<0.001
DHA C22:6 n3	15.98 ± 0.54^a^	12.31 ± 0.52^b^	6.17 ± 0.60^c^	1.41 ± 0.14^d^	<0.001	<0.001	0.270
Total n3	386.54 ± 1.49^a^	315.35 ± 2.65^b^	190.52 ± 0.53^c^	39.93 ± 0.47^d^	<0.001	<0.001	<0.001
n6:n3	1.35 ± 0.00^d^	1.79 ± 0.02^c^	3.10 ± 0.01^b^	14.86 ± 0.17^a^	<0.001	<0.001	<0.001

*Note*: A = 30 days, B = 20 days, C = 10 days, D = 0 days; SFA = saturated fatty acid, MUFA = mono-unsaturated fatty acid, ETA = Eicosatetraenoic acid, EPA = Eicosapentaenoic acid, DHA = docosahexaenoic acid; data are presented as mean; n = 6. Means in the same column are significantly different at *α* = 0.05.

**Table 9:** Effect of supplemental different days of flaxseed on fatty acid profile of skin of Peking ducks

Supplemental days	30	20	10	0	*P*	Linear	Quadratic
Fatty acids (mg/100 g)							
Myristic acid C14:0	38.43 ± 0.33^d^	47.27 ± 0.71^c^	57.98 ± 1.38^b^	87.72 ± 0.68^a^	<0.001	<0.001	<0.001
palmitic acid C16:0	284.82 ± 0.44^d^	296.93 ± 0.92^c^	308.48 ± 0.52^b^	369.61 ± 1.71^a^	<0.001	<0.001	<0.001
Margaric acid C17:0	50.98 ± 0.38^d^	54.84 ± 0.35^c^	64.33 ± 2.08^b^	89.51 ± 0.77^a^	<0.001	<0.001	<0.001
Total SFA	374.24 ± 0.63^d^	399.04 ± 1.09^c^	430.79 ± 3.05^b^	546.84 ± 1.50^a^	<0.001	<0.001	<0.001
Palmitoleic acid C16:1	395.71 ± 1.21^d^	403.71 ± 1.52^c^	429.10 ± 1.23^b^	437.50 ± 0.59^a^	<0.001	<0.001	0.871
Oleic acid C18:1n9c	379.14 ± 2.74^d^	415.87 ± 1.43^c^	456.23 ± 2.02^b^	489.56 ± 0.70^a^	<0.001	<0.001	0.378
Arachidic acid C20:0	28.47 ± 0.89^d^	46.37 ± 0.71^c^	53.64 ± 0.61^b^	63.43 ± 1.66^a^	<0.001	<0.001	0.001
Total MUFA	803.33 ± 1.83^d^	865.94 ± 3.37^c^	938.97 ± 2.71^b^	990.49 ± 1.25^a^	<0.001	<0.001	0.033
Linoleic acid C18:2n6	446.65 ± 0.63^c^	471.53 ± 0.38^bc^	479.91 ± 16.24^ab^	505.67 ± 7.38^a^	<0.002	<0.001	0.961
Ecosadienioc acid C20:2n6	13.51 ± 0.11^d^	14.52 ± 0.07^c^	16.56 ± 0.09^b^	18.30 ± 0.07^a^	<0.001	<0.001	0.027
Dihomo-γ-linolenic acid C20:3n6	73.73 ± 0.55^c^	76.69 ± 0.59^b^	80.74 ± 0.89^a^	77.95 ± 0.67^b^	<0.001	<0.001	<0.001
Arachidonic acid 20:4n-6	10.91 ± 0.15^b^	12.66 ± 0.07^a^	12.98 ± 0.39a	12.42 ± 0.10^a^	<0.001	<0.001	<0.001
Total n 6	544.81 ± 0.86^c^	575.41 ± 0.44^b^	590.19 ± 16.36^ab^	614.33 ± 7.84^a^	<0.001	<0.001	0.726
Linolenic acid n3	358.78 ± 3.43^a^	288.15 ± 2.18^b^	168.88 ± 2.81^c^	40.36 ± 0.21^d^	<0.001	<0.001	<0.001
ETA C20:4 n3	11.94 ± 0.34^a^	7.06 ± 0.26^b^	4.19 ± 0.35^c^	0.45 ± 0.04^d^	<0.001	<0.001	0.053
EPA C20:5 n3	19.10 ± 0.47^a^	17.94 ± 0.32^b^	10.65 ± 0.31^c^	1.34 ± 0.05^d^	<0.001	<0.001	<0.001
DHA C22:6 n3	18.90 ± 0.48^a^	11.76 ± 1.98^b^	5.92 ± 0.32^c^	0.78 ± 0.04^d^	<0.001	<0.001	0.343
Total n3	408.73 ± 2.77^a^	324.92 ± 3.60^b^	189.64 ± 3.48^c^	42.93 ± 0.24^d^	<0.001	<0.001	<0.001
n6:n3	1.33 ± 0.01^d^	1.77 ± 0.02^c^	3.11 ± 0.03^b^	14.31 ± 0.11^a^	<0.001	<0.001	<0.001

*Note*: A = 30 days, B = 20 days, C = 10 days, D = 0 days. SFA = saturated fatty acid, MUFA = mono-unsaturated fatty acid, ETA = Eicosatetraenoic acid, EPA = Eicosapentaenoic Acid, DHA = docosahexaenoic acid.Data presented as mean; *n* = 6. Means in the same column are significantly different at *α* = 0.05.

## Discussion

Flaxseed is known to be the richest source of n-3 fatty acid among the terrestrial sources of n-3 PUFA. It contains seven times more ALA than soybean and corn oil with three times less LA content (35). The marine or preformed sources of n-3 are fish oil and microalgae and their supplementation increase DHA in the eggs threefold as compared to flaxseed oil but it gives fishy flavor to the products (36, 37). Flaxseed supplementation in ducks increased the number of bacteria causing inflammation but this was diminished with prolonged duration of flaxseed feeding (38). Previous reports have shown the poor growth performance of birds fed with flaxseed (20). In this study, ducks’ growth performance was negatively affected when fed with flaxseed at 10% for 30 days, most likely due to the presence of anti-nutritional factors such as linatine and non-starch polysaccharides (NSP) in flaxseed mucilage (39). This is because poultry lack enzymes to break the bonds between sugars in NSP (40), which could lower the digestion and availability of nutrients (41). Hence, the use of enzymes can be helpful to degrade NSP and alleviate the negative effect of flaxseed on growth performance in poultry (19). Previous studies report that increased LPL is associated with reduced fat accumulation in the body by regulating endogenous fatty acid oxidation which could reduce body weight (42, 43). Moreover, LIPIN-1 mRNA expression in chicken has also shown to decrease fat deposition (44). In this study, flaxseed increased the LPL and LIPIN-1 gene expression which could reduce the abdominal fat deposition and could ultimately be responsible for reduced body weight gain in the ducks.

The relative weight of skin and abdominal fat decreased linearly with increasing duration of the flaxseed diet in agreement with previous results of linseed oil fed broilers (6, 20). In our study, increasing the duration of the flaxseed diet resulted in a linear increase of good cholesterol (HDL) and a linear decrease in bad cholesterol like LDL, VLDL, and TG. This is in agreement with results indicating that diet enrichment with PUFA has a beneficial effect on serum lipid profile (45) or that increasing n-3 PUFA in the diet of chickens increases HDL and reduces TG (46). This reduction may be related to the role of n-3 FA in the suppression of TG, apolipoprotein synthesis, higher elimination of VLDL by peripheral tissues of the liver and higher excretion of bile via feces (47), resulting in a reduction in cholesterol and TG concentrations in serum.

High-fat diets increase the serum LPS activity by increasing permeability of the intestine through inhibition of the protein expression from the tight junctions, allowing LPS molecules to enter from the intestine into circulation (48–50). In our study, LPS activity increased with the duration of the flaxseed diet. It is believed that HDL helps in LPS detoxification but its binding with HDL makes it difficult to clear from circulation (51). In our study, LPL enzymatic activity increased linearly with the duration of the flaxseed diet. LPL activity in animal tissues increases with the consumption of diets rich in PUFA (52). The enzymatic activity of LPL represents a limiting step for the entry of dietary fatty acid into tissues because LPL hydrolyzes circulating TG in the form of porto microns and very-low-density lipoproteins in order to enter the tissues (53).

Fatty acid manipulation via dietary means may provide an effective way to obtain n-3 enriched animal products for human consumption. Flaxseed oil and full-fat flaxseed are valuable sources of ALA in chicken diets and effectively incorporated from feed to the bird’s tissues (54). Dietary flaxseed oil significantly increased n-3 PUFA concentrations and decreased n-6:n-3 PUFA ratios in birds fed diets containing soybean oil (55). In our study, total n3 increased linearly in breast muscle and skin, while SFA, MUFA, and n-6 PUFA linearly decreased with the duration of the flaxseed diet.

In poultry, *de novo* lipogenesis occurs in the liver from dietary carbohydrates. These reactions are catalyzed by glucose-6-phosphate, dehydrogenase, and malic enzyme (56). The conversion of oil sources rich in C18:3 into C20:5 and C22:6 had been reported previously (57). However, in our experiment, n-3 content in breast muscles and skin increased with the duration of the diet. C20:5 and C22:6 FA were observed in breast and skin, indicating hepatic elongation and desaturation of FA. The increase in omega-3 in breast muscle and its conversion to EPA and DHA with increased duration of the diet might be due to the higher hepatic expression of the FADS2 gene, which competes for both n-3 and n-6 (7).

The liver is the major site of lipogenesis and de novo fatty acid biosynthesis as the liver transfers lipids to other tissues through blood and exerts an effect on the lipid content and composition (58). LPIN1, LPIN2, WDTC1, and FADS2, are lipid metabolism genes with vital functions in fat metabolism (6). LPIN1 is an enzyme, involved in the de novo biosynthesis of TG and its mRNA is abundant in adipose tissue and muscle tissue of mice (59). In our study, feeding flaxseed to ducks for 30 days upregulated mRNA expression of LPIN1 (10). Lipin1 can also act as a transcriptional co-activator in relation to peroxisome proliferator-activated receptor alpha (PPAR-α). These are nuclear receptors that act as major regulators of hepatic lipid catabolism, targeting gene expression associated with inflammation, glucose metabolism, and most importantly, the lipid-related pathway (60). Lipin-1 enhanced expression of PPAR-α target genes with direct interaction with PPAR receptors (61). In our study, increased expression of Lipin-1 activated PPAR-α expression, but not significantly, indicating that Lipin-1 can co-activate PPAR-α.

FADS2 expression increased at 10 and 30 days while at 20 days its expression was still above that of the control diet. A previous study (28) also reported that flaxseed oil increased expression of FADS2 at day 7 of feeding prior to slaughter, but FADS2 decreased when the duration of the flaxseed diet increased. Jing et al. (9) suggested that FADS2 gene expression changed over time according to the dietary n-6:n-3 ratio, supporting the concept that metabolic enzymes of long-chain PUFA are modulated by dietary fatty acid composition. It was proposed in the previous studies that consumption of an n-3 enriched diet could stimulate mRNA expression of FADS2, due to the higher affinity of FADS2 for n-3 than for n-6 (62, 63).

WDTC1 is an obesity-linked gene which appears to inhibit fat formation in a dosage-sensitive manner in both animals and humans (64). In this study, feeding flaxseed had no effect on WDTC1 gene expression. FATP, proposed as a major FA transporter in intestinal lipid absorption, is necessary for the transportation of long-chain FA across the plasma membrane and for esterification (5). L-FABP played a key role in transporting FA through the cytosol of absorptive cells (65). Long-chain polyunsaturated FA had a higher affinity towards L-FABP (66). In our study, there was no difference in expression of L-FATP and FABP between control and flaxseed fed ducks, which could be due to the high level of n-6 PUFA in the control group diets and breast muscle. Over-expression of LPIN1 promotes fat deposition in mice (44) but decreases fat deposition in chicken (67). In addition, high hepatic PPAR-α expression reduced abdominal fat accumulation in broilers (68). We found that an increase in LPIN 1 gene expression reduced abdominal fat deposition and that PPAR-α expression did not change significantly but was higher after 30 days of feeding flaxseed, compared to the control diet, resulting in reduced abdominal fat in the Peking ducks.

In our study, the observed decrease in the lipid oxidation in the meat, liver, and jejunal mucosa, might be related to the antioxidant activity of vitamin E in the premix added to the diets. The contents of vitamin E in the body could enhance total antioxidant activity. AST and, especially, ALT, are good indicators of liver damage. High levels of liver enzymes in serum are usually due to hepatocytes necrosis and altered membrane permeability (69). In the current study, flaxseed with vitamin E decreased hepatic enzymatic activity like AST in serum. The improvement in serum’s AST could be due to the beneficial effect of vitamin E on duck liver. The drip loss (water-holding-capacity) of meat depends on intramuscular lipids, moisture content, and lipid peroxide content in the muscle (70). Flaxseed supplementation resulted in an increase in the oxidation of breast meat (71). We found that the drip loss of breast meat decreased with the duration of the flaxseed diet. This improvement in drip loss might be due to the antioxidant capacity of vitamin E, which could lower the lipid oxidation in the breast muscle, enhance antioxidant enzymes’ activities, enhance the integrity of cellular membranes, and thereby decrease drip loss in the muscle (72).

From the economic perspective, the price of flaxseed is approximately twice the price of wheat and corn. In order, to get maximum profit from the supplemented flaxseed feeding duration to get duck meat enriched with n-3 PUFA, a threshold level is required for labeling this meat as n-3 enriched duck meat. According to the Canadian Food Inspection Agency, 2003, the threshold level of 300 mg/100 g of total n-3 fatty acid of meat is required for labeling it as n-3 FA enriched (73). However, in this study, increasing the duration of feeding a flaxseed diet had mildly decreased BW and increased FCR which ultimately elevated the cost of production. On the other hand, feeding flaxseed for the 20 days’ duration potentially enriched the duck breast muscle with n-3 PUFA (386.54 mg/100 g of meat) before the completion of the flaxseed-feeding duration (30 days). A feeding duration of 20 days before slaughtering is optimal to attain a threshold level of n-3 PUFA in the Peking duck meat which would lower the cost of production for commercial use. It is worthy of attention to note that n-3 enriched meat and eggs are already popular in the world, and health-conscious consumers are willing to pay a premium price for them (74). Use of carbohydrase enzyme may reduce the negative effect of flaxseed in poultry (75), which could increase the palatability of flaxseed and also improve the n-3 enrichment efficiency for health-conscious consumers.

## Conclusion

Feeding flaxseed with vitamin E for a period of 30 days had a mild adverse effect on the performance of ducks but enhanced meat quality and decreased lipid oxidation in meat. The feeding period of 20 days before slaughtering is adequate to enrich meat with EPA and DHA and decrease the n-6 to n-3 ratio. The enrichment of duck meat with long-chain PUFA (EPA and DHA) by using flaxseed is appropriate for health-conscious consumers and could replace the marine sources of EPA and DHA in the diet of poultry. Further studies are suggested to investigate the effect of flaxseed on the fatty acid profile of raw and cooked poultry meat.
